# Oscillatory ventilation enhances oxygenation and reduces inflammation in an animal model of acute respiratory distress syndrome: an experimental study

**DOI:** 10.1016/j.bjane.2024.844576

**Published:** 2024-11-24

**Authors:** Luiz Alberto Forgiarini Junior, Luiz Felipe Forgiarini, Arthur de Oliveira Paludo, Rodrigo Mariano, Mikael Marcelo de Moraes, Elaine Aparecida Felix, Cristiano Feijó Andrade

**Affiliations:** a Universidade Católica de Pelotas (UCPel), Pelotas, RS, Brasil; b Centro Universitário Ritter dos Reis (Uniritter), Porto Alegre, RS, Brasil; c Universidade Federal do Rio Grande do Sul (UFRGS), Faculdade de Medicina, Programa de Pós-Graduação em Ciências Pneumológicas, Porto Alegre, RS, Brasil; d Hospital de Clinicas de Porto Alegre (HCPA), Porto Alegre, RS, Brasil

**Keywords:** Acute respiratory distress syndrome, Mechanical ventilation, Positive-pressure respiration

## Abstract

**Background:**

This study aims to compare the use of variable mechanical ventilation with conventional mechanical ventilation in a porcine model of ARDS induced by oleic acid.

**Methods:**

The animals were divided into two groups (n = 6), Conventional Ventilation (CO) and variable ventilation with Bi-Oscillatory PEEP (BiPEEP). ARDS was induced using intravenous oleic acid (0.15 mL.kg^−1^). After, the animals were evaluated during 180 minutes and, measurements were taken every 30 minutes until the end of the observation period. The animals in the CO group were then ventilated under controlled pressure (Tidal Volume target at 6 mL.kg^−1^) and 5 cm H_2_O PEEP. Variable ventilation was characterized by the oscillation of PEEP from 5 to 10 cm H_2_O every 4 respiratory cycles. Ventilatory, hemodynamic parameters, oxidative stress, antioxidant enzymes, Interleukin 8 (IL8) and 17-a (IL17a) were evaluated. Histological samples were collected from the upper and the lower portion of the left lungs and analyzed separately.

**Results:**

BiPEEP improved lung compliance and PaO_2_ in comparison to control (p < 0.05). The levels of oxidative stress and antioxidant enzymes showed no significant difference. There was no difference in IL17a between groups. IL8 was significantly increased in the lung base of CO group in relation to BiPEEP group and it was reduced in the apex of BiPEEP group in comparsion to CO group. The BiPEEP group showed less changes in histopathological patterns.

**Conclusion:**

Variable ventilation with bi-oscillatory level of PEEP demonstrated a potential ventilatory strategy for lung protection in an experimental model of ARDS.

## Introduction

Acute Respiratory Distress Syndrome (ARDS) is characterized by acute hypoxemia (PaO_2_/FiO_2_ < 300 mmHg) and the presence of bilateral infiltrates on chest X-Ray, not explained by the presence of left atrial hypertension, and may be associated with severe hypoxemia.^1^

In the literature, there is great diversity regarding how to ventilate patients with ARDS, but the most used approaches are based on either the ARDS Network or the Open Lung Approach (OLA).^2^^,^^3^ However, if there is a down adjustment of the values of Positive end Expiratory Pressure (PEEP), this may result in alveolar derecruitment, and similarly high levels of PEEP may be associated with stress and over-distension of the pulmonary parenchyma.^4^

Different ventilatory alternatives have been proposed in patients with ARDS to protect the lungs, improve oxygenation and eventually reach better outcomes.^5^^,^^6^ Nonetheless, most of these ventilatory strategies differ from spontaneous ventilation because they have a pre-stablished ventilatory pattern, characterized by the maintenance of the respiratory rate, tidal volume and a fixed pressure ventilation.^7^ In this setting, variable ventilation has been suggested to improve lung function in experimental models of ARDS and could be a very interesting and effective way to ventilate injured lungs.^8^ Different approaches with satisfactory results have been proposed for the use of variable ventilation such as changes in respiratory rate, tidal volume and pressure support.^9^, ^10^, ^11^, ^12^ Therefore, in variable ventilation one of the ventilatory variables is not fixed or constant. Conventional mechanical ventilation is considered monotonous because the variables are predominantly fixed and constant. Unfortunately, during the pursuit for the ideal ventilatory strategy we face the dilemma between collapse and overdistention. One has to choose an adequate PEEP to prevent collapse during expiration, but at the same time, it has to be low enough to prevent overdistension of previously aerated lung regions. In this study we hypothesized that oscillatory PEEP (BiPEEP) in an experimental model of ARDS is capable of improving gas exchange and reducing inflammatory response when compared to monotonous ventilation.

## Methods

The study complies with the regulations of the Brazilian Code of Practice for the care and use of animals for scientific purposes and was approved by the Animal Ethics Committee of the Hospital de Clínicas de Porto Alegre (Protocol 11-432). The present study was carried out in accordance with ARRIVE Guidelines (https://www.equator-network.org/reporting-guidelines/).

### Preparation of animals, anesthesia and ventilation

In this study we used 12 male pigs (Large White), weighing on average (SD) 25 ± 13 kg in a protocol of lung injury induced by oleic acid. The allocation of animals to groups was carried out through the website www.randomization.com. The animals were divided into two groups (n = 6), ARDS group with monotonous ventilation (Control) and an oscillatory PEEP group (BiPEEP). Following intramuscular sedation (ketamine 10 mg.kg^−1^ and midazolam 1 mg.kg^−1^) anesthesia was maintained with propofol, morphine, and ketamine as previously described.^12^

The pigs were mechanically ventilated (Inter 7 Plus, Intermed Equipamentos Médicos Hospitalares Ltda, São Paulo, Brasil) via a size 8 mm ID (Internal Diameter) endotracheal tube using the following settings: pressure control, FiO_2_ 1.0, inspiration to expiration ratio = 1:2, target tidal volume 6 mL.kg^−1^ with the initial respiratory rate adjusted to maintain an end tidal CO_2_ tension of 35 to 45 mmHg. The ventilatory parameters were adjusted to maintain a plateau pressure limit of 35 cm H_2_O. With the exception of PEEP, the ventilation settings were not changed during the entire protocol. The PEEP setting was 5 cm H_2_O in the control group. In the oscillatory bi-level PEEP group, the animals were ventilated with the same ventilatory pattern with the exception of PEEP, which was automatically increased to 10 cm H_2_0 every 4 breaths.

### ARDS injury

After a set of baseline measurements, ARDS was performed by injection of 0.15 mL.Kg^−1^ oleic acid (Sigma-Aldrich®, Steinheim, Germany) in 15 mL of saline administered over 10 minutes through the proximal end of the pulmonary artery catheter^13^ until a P/F ratio of 200 to 300 mmHg. Intravenous noradrenaline was used to maintain a MAP ≥ 70 mmHg during the infusion of oleic acid. Intravenous fluid administration was limited to 1 mL.kg^−1^.h^−1^, after an initial 500 mL over a 30-minute bolus of succinylated gelatin.

### Experimental protocol

All 12 pigs received oleic acid. Analysis of respiratory mechanics, ventilatory parameters and arterial blood gases were performed after stabilization of the animals (post-induction). After, the animals were evaluated for 180 minutes and during this period measurements were made every 30 minutes until the end of the observation period. At the end of the experiment, the animals were sacrificed by exsanguination, and we collected tissue samples from the upper and lower portion of the left lungs.

### Respiratory mechanics and lung volumes

For the evaluation of mechanical properties of the respiratory system and measures of airflow we used a flow transducer, located in the proximal portion of the endotracheal tube, the Monitor Graph Ventilation – Tracer 5® (Intermed Ltda – São Paulo, Brazil) on the computer which recorded Pressure curves (P), flow (V ') and Volume (V) versus time (t); curve pressure × volume curve (compliance) and × flow volume curve, through specific software.

Arterial oxygen tension (PaO_2_), oxygen saturation, and carbon dioxide tension were measured with a blood gas analyzer immediately after withdrawal (Rapidlab 1200, Siemens, Leverkusen, Germany). PaO_2_ over fractional inspiratory oxygen concentration (P/F ratio) was calculated.

### Hemodynamic parameters

Mean Arterial blood Pressure (MAP) was measured at the femoral artery. Throughout the study the animals remained in supine position. All hemodynamic pressures were zeroed at the mid axillary line at the level of the sternum and measured during end-expiration.

### Oxidative stress – assessment of lipid peroxidation

The Thiobarbituric Acid Reactive Substances (TBARS) technique consists of heating the homogenate with thiobarbituric acid to produce a colored product that is subsequently measured at 535 nm using a spectrophotometer. The TBARS concentration was expressed in nmoL.mg^−1^ of protein.^14^

### Antioxidant enzymes – determination of superoxide dismutase (SOD)

The technique used to measure SOD was based on the level of inhibition caused by the reaction of the enzyme with O^−2^. The enzymatic activity was expressed in SOD/mg of protein units.

### Interleukin analysis

After the samples were thawed, a 96-well plate was coated with monoclonal antibodies against IL-8 and IL-17a. The IL-8 and IL-17a concentrations in the homogenized lung samples were calculated based on the results of a standard curve.

### Histological analysis

The lung tissue specimens were fixed in formalin and dehydrated, cleared, and embedded in paraffin. The specimens were cut into 5-μm serial sections and stained with hematoxylin-eosin. The same regions were sampled in all groups. A pathologist blinded to the experimental protocol and the region of sampling performed quantitative analysis by light microscopy. Each sample was examined under both low- and high-power fields. At least four sections were obtained from each block, and 20 fields were randomly selected and analyzed for each section. The severity of histological lesions was assessed using a histologic score^15^ based on six parameters: intraalveolar edema, hyaline membrane formation, hemorrhage, recruitment of granulocytes into the air spaces, focal alveolar collapse or consolidation, and epithelial desquamation or necrosis of the airways or alveoli. Each parameter was evaluated semi quantitatively using the following scale: 0, absent; 1, mild; 2, moderate; and 3, prominent. In addition, the percentage of the involved area of each histological specimen was estimated (0- = -100%) to quantify the histological changes.^16^

### Statistics

The sample size calculation with a confidence interval of 95% for the experimental groups was performed. Normal distributions of means tests were performed for statistical tests. The groups were compared using analysis of variance (ANOVA) followed by a post hoc Tukey test performed using SPSS® version 19.0 (Statistical Package for Social Science). In the case of unequal variances or an abnormal distribution, a nonparametric Kruskal Wallis test was performed, followed by Mann-Whitney *U*-tests for intergroup comparisons. The results are represented as means ± standard deviation. The statistical significance level was set at p < 0.05.

## Results

There were no significant differences between the groups in terms of mechanical ventilation, with exception for airway resistance from Baseline to 30 minutes in the Control group (Table 1). The groups were not different at baseline, and similar changes in heart rate, PAP and end tidal CO_2_ increased in both groups after OA infusion (Table 2).

**Table 1:** Comparison between groups in relation to ventilatory variables.

	Baseline		Post-induction		30 minutes		60 minutes		90 minutes		120 minutes		150 minutes		180 minutes	
	Control	BIPEEP	Control	BIPEEP	Control	BIPEEP	Control	BIPEEP	Control	BIPEEP	Control	BIPEEP	Control	BIPEEP	Control	BIPEEP
Ppico	17 (15‒17.2)	17 (17‒18.2)	24 (19.2‒24.7)	17 (17‒23.7)	25 (19.2‒27)	21 (19‒25.5)	26 (19.2‒27.5)	24 (20.7‒30)	25 (19.2‒27.7)	27 (23‒31)	26 (18.5‒27.5)	23 (19.5‒26)	26 (18‒27.7)	24 (23‒26)	26 (18.5‒27.7)	25 (23‒27.2)
Pplatô	15 (13.5‒15.2)	17 (14‒15.5)	20 (15.2‒21.2)	14 (14‒20)	22 (15.5‒22)	16 (14‒20.2)	21 (15.5‒22.5)	20 (17.5‒22.5)	22 (15.5‒23)	21 (19‒26.5)	22 (14.7‒23)	22 (16.7‒27.2)	22 (14.7‒22.5	20(17.5‒25)	22(14.7‒22.5)	20 (19‒24.2)
VACe	272 (231‒399)	225 (212‒373)	210 (188‒254)	206 (185‒258)	222 (205‒227)	212 (184‒256)	210 (185‒238)	270 (194‒403)	198 (155‒214)	270 (191‒416)	197 (157‒221)	290 (184‒471)	194 (184‒223)	270 (190‒483)	199 (192‒231)	265 (195‒481)
Resist	19.9±9.6	25.9±3.6	26.3±11	30.6±5.7	35.8±6.4	33.5±8	30.1±3.7	31.8±7.0	30.8±3.7	32.8±4.9	30.8±4.3	26.2±5.2	29.8±3.3	30.1±2.3	29.9±3.3	29.1±24
Cest	29.4±8.5	23.8±7.1	13.9±3.1	15.9±8.4	12.9±2.8	14.1±6.7	13.7±3.5	16.1+8.3	12.9±3.4	18.6±11.5	26±3.5	26.2±5.2	12.2±3.2	17.6±10.4	12.6±3.1	19.5±93
Cdin	25.3±4.5	24.3±6.3	10.9±3.1	11.4±4.8	9.8±2.1	10.8±5.1	10±1.9	11.5±5.1	10.2±2.8	14.3±9.2	10±2.9	14.2±9.3	9.7±2.5	14.7±8.1	9.8±2.4	15.8±86

Mechanical ventilation parameters: PPeak, Peak Pressure; Plateau, Plateau pressure; VT, Tidal Volume; Resist, Airway Resistance; Cest, Static Compliance; Cdin, Dynamic Compliance. Make the difference between Baseline Control Resist with 30-minutes Control Resist. Results were expressed in median and interquartile range our mean and standard deviation.

**Table 2:** Comparison between groups in relation to hemodynamic variables.

	Baseline		After induction		30 minutes		60 minutes		90 minutes		120 minutes		150 minutes		180 minutes	
	PEEP	BIPEEP	PEEP	BIPEEP	PEEP	BIPEEP	PEEP	BIPEEP	PEEP	BIPEEP	PEEP	BIPEEP	PEEP	BIPEEP	PEEP	BIPEEP
HR	79±18	83.2±20.3	92±21.9	99±31.4	95.6±21.3	88.6±18.3	88.6±18.3	103.6±34	87.8±18.4	108.4±30.3	95.6±21.1	111±34.9	91.6± 13	108.6± 35.1	103±5.8	103.8±22.4
PAM	105.2±20.9	86.8±5.2	93.2±16.8	99±5.6	87.6±12.2	98±8.9	92.2±11.5	92.4±12.3	93.7±8.7	94.6±10.5	87.5±12.4	95.6±14.5	95.2± 15.4	91.8± 8.5	91.5±19.3	93.7±8.5
PAP	29.4±10.9	25.6±4.1	41±11.3	40±7.8	33±10.8	35±9.7	35±10.8	33.2±12.2	39.8±12.4	33±15.4	35.8±12.2	36.6±14.6	35.8± 10.2	35± 15.5	35.8±10.5	36.5±14.6
PCP	17.2±8.3	13.2±2.3	15±5.3	15.2±4.6	14.5±2.3	13.6±3	15±2.6	14±1.2	15±2.9	13.8±1.3	15±2.5	11.2±2.9	11.3± 9.8	11.4± 2.2	15±2.6	11.4±2.2
ETCO_2_	37±10.3	38.2±9.6	47±4.7	52.4±12.3	52.2±4.8	51.3±13.1	48±7.4	50.7±10.3	51.8± 5.9	52.1±9.6	49.8±4.9	51.9±7.4	51.6± 5.8	49.8+9.8	52.2±5.6	51.1±7.8

HR, Heart Rate; MAP, Mean Arterial Pressure; PAP, Pulmonary Arterial Pressure; PCP, Pulmonary Capillary Pressure.

**Figure 1:**
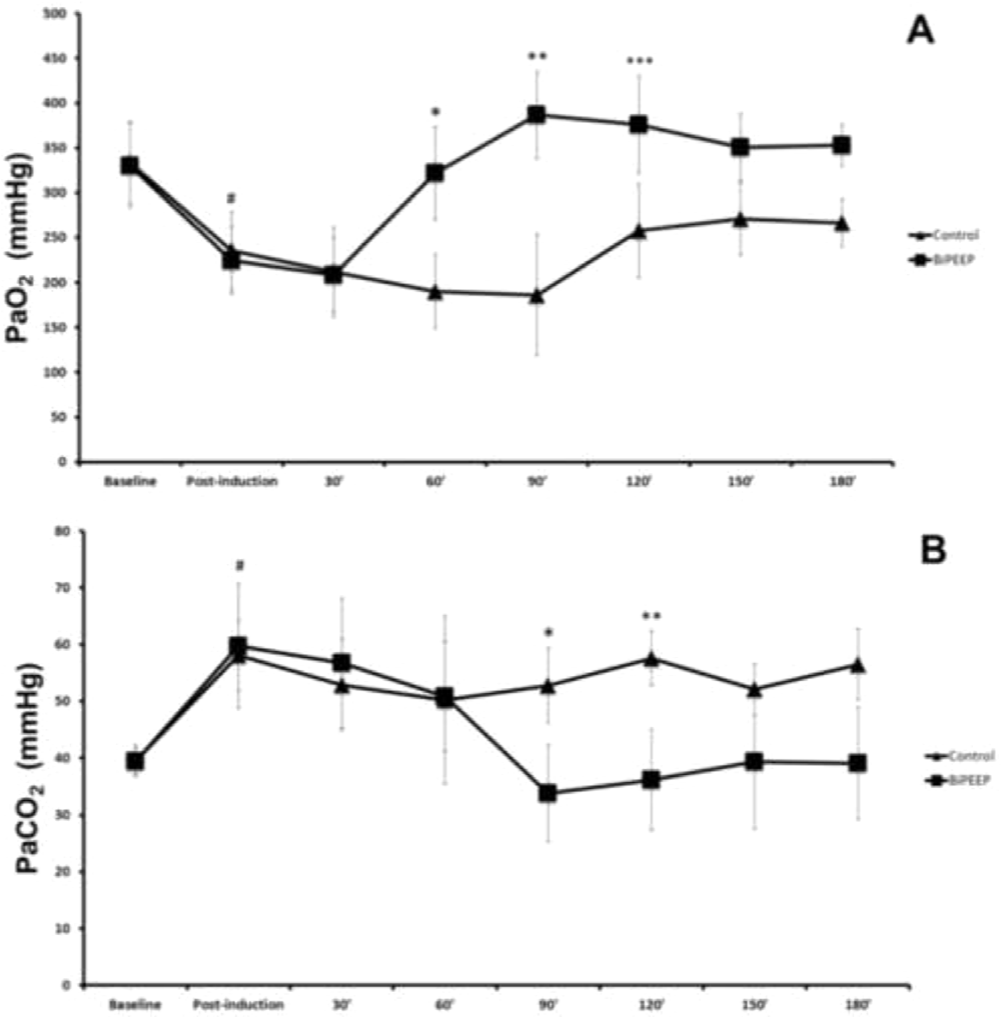
Arterial pressure of Oxigen (PaO_2_) in Blood gas analyses (A). Demonstrate significantly difference between Baseline groups with Post-induction groups (# p < 0.05), 60’ Control with 60’ BiPEEP (* p < 0.001), 90’ Control with 90’ BiPEEP (** p < 0.001) and 90’ Control with 90’ BiPEEP (*** p < 0.01). Arterial pressure of Dioxide Carbon (PaCO_2_) in Blood gas analyses (B). Make the difference between Baseline groups with Post-induction groups (# p < 0.05), 90’ Control with 90’ BiPEEP (* p < 0.05) and 120’ Control with 120’ BiPEEP (** p < 0.01). Six animals per group.

There was a significant difference in arterial oxygen pressure (PaO_2_) at baseline in the control group when compared to post-induction control. The same results were observed at baseline in the BiPEEP group when compared to post-induction BiPEEP (p < 0.05). The PaO_2_ significantly dropped after induction and remained at low levels in both groups. The fall in PaO_2_ was significantly greater in the control group (Fig. 1A).

The blood pressure of carbon dioxide (PaCO_2_) was significantly lower at baseline in the control group when compared to post-induction control (p < 0.05). The same finding was observed in the BiPEEP group. In the control group at 90 (p < 0.05) and 120 (p < 0.01) minutes, PaCO_2_ was significantly higher compared to the BiPEEP group. At times 90 (p < 0.001), 120 (p < 0.01), 150 and 180 minutes (p < 0.05) BiPEEP significantly reduced PaCO_2_ values when compared to post-induction BiPEEP (Fig. 1B).

**Figure 2:**
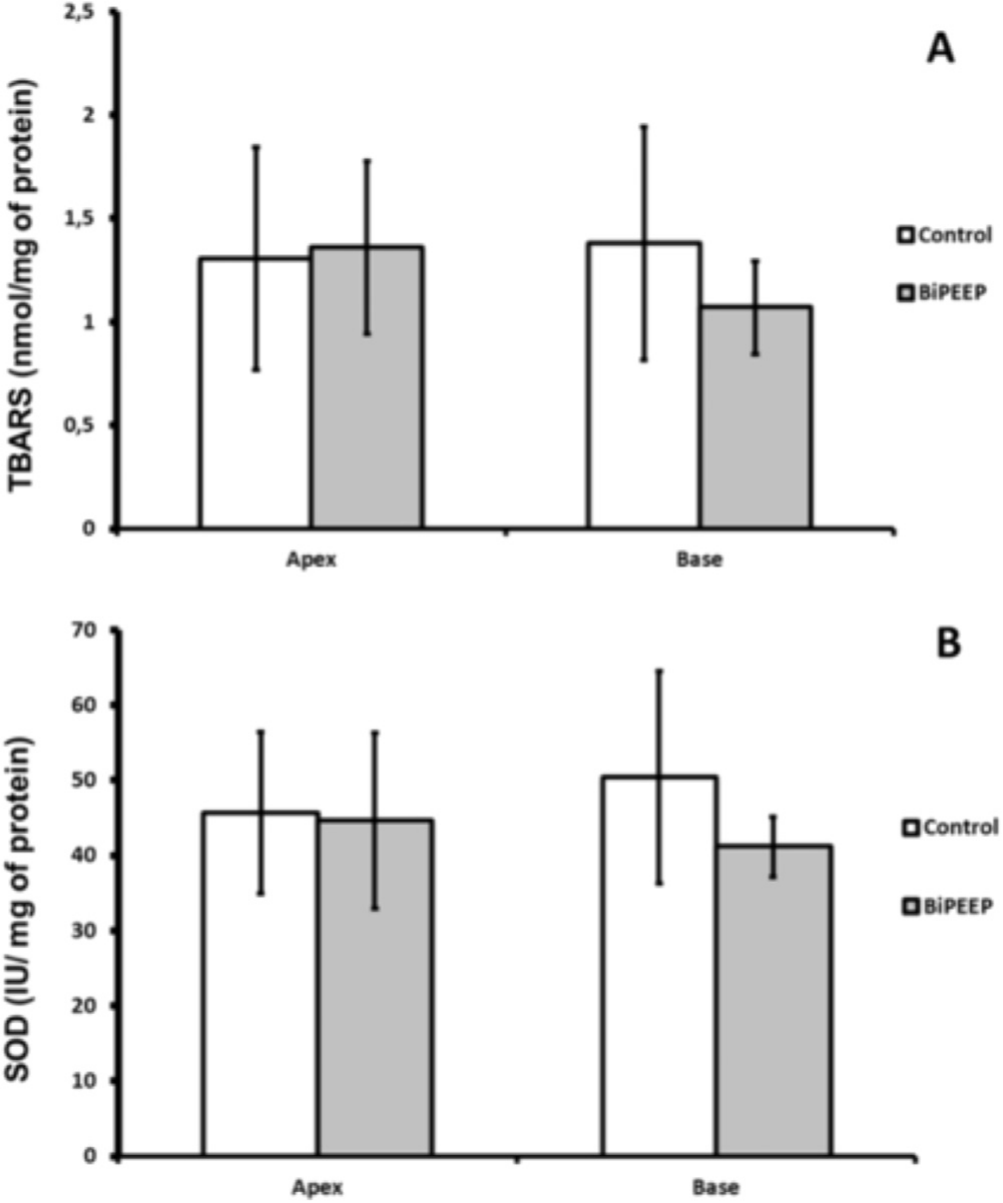
Oxidative Stress and antioxidant enzymes. There was not significant difference between groups at the quantity of lipid peroxidation products (A). There was no difference shown in the SOD activity among the groups (B). Six animals per group.

There were no significant differences between groups and lung regions as to the number of lipid peroxidation products (Fig. 2A). The activity of superoxide dismutase did not change significantly between both groups (Fig. 2B).

**Figure 3:**
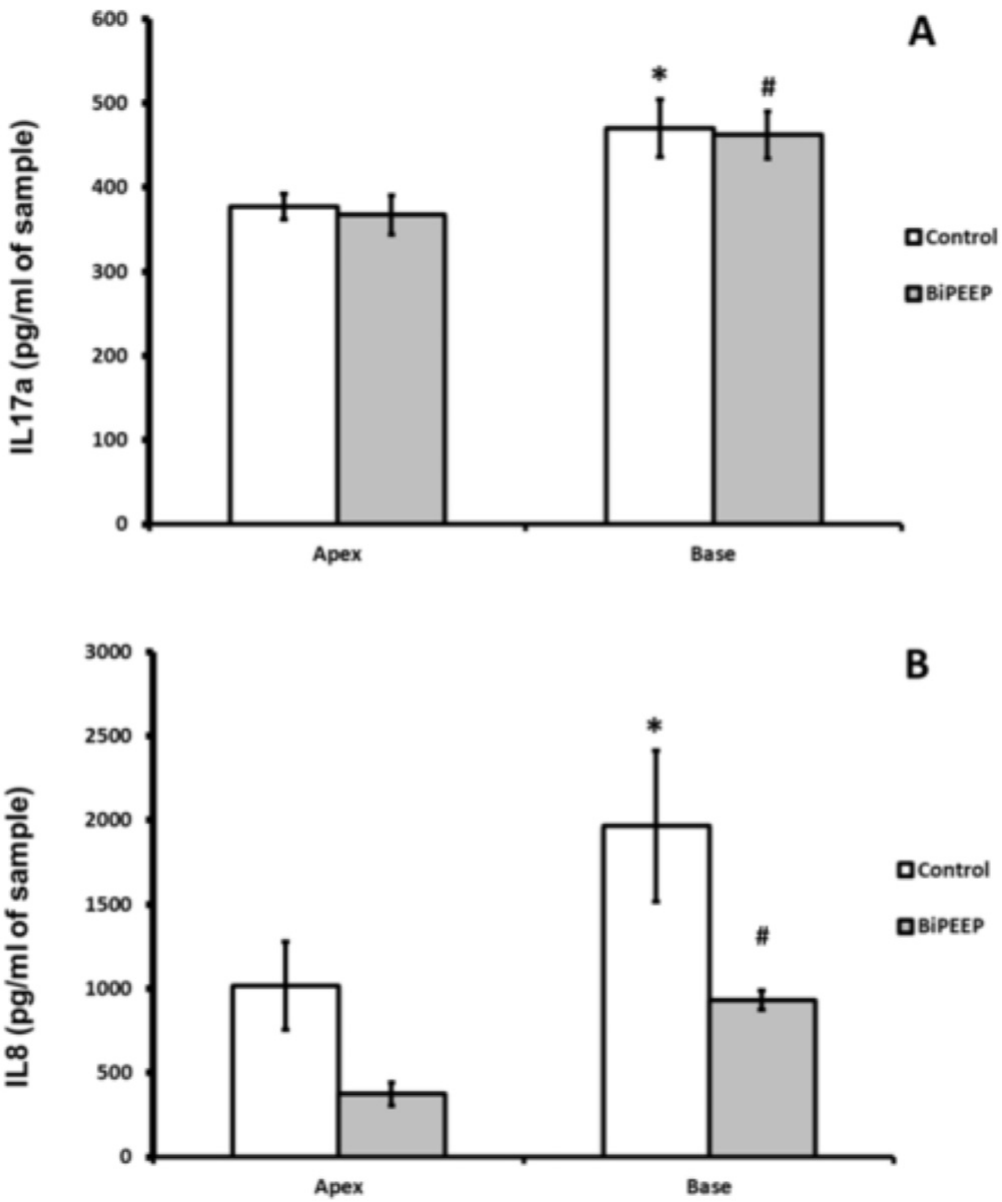
The Interleukin 17a (IL17a) expression (A) demonstrates a significantly difference between apex and base of each group (* p < 0.01 and # p < 0.01). Interleukin 8 (IL8) expression (B) increased in the both groups between apex in relation to the base (p < 0.01). The difference was observed to the Control base in relation to BiPEEP base (* p < 0.001) and apex intergroups (# p < 0.01). Six animals per group.

Interleukin-17a (IL-17a) showed no significant difference between the control and BiPEEP groups. There was a significant difference (p < 0.01) between the apex and the base of lung samples of each group (Fig. 3A). Interleukin-8 (Fig. 3B) demonstrated a significant increase in the samples of lung base from the control group when compared to the BiPEEP group (p < 0.001). The apex of the lung in the BiPEEP group presented a significant reduction in IL8 in comparison to control (p < 0.01). In both groups there was a significant difference in IL-8 concentration in the apex when compared to the base of the lungs (p < 0.01).

**Figure 4:**
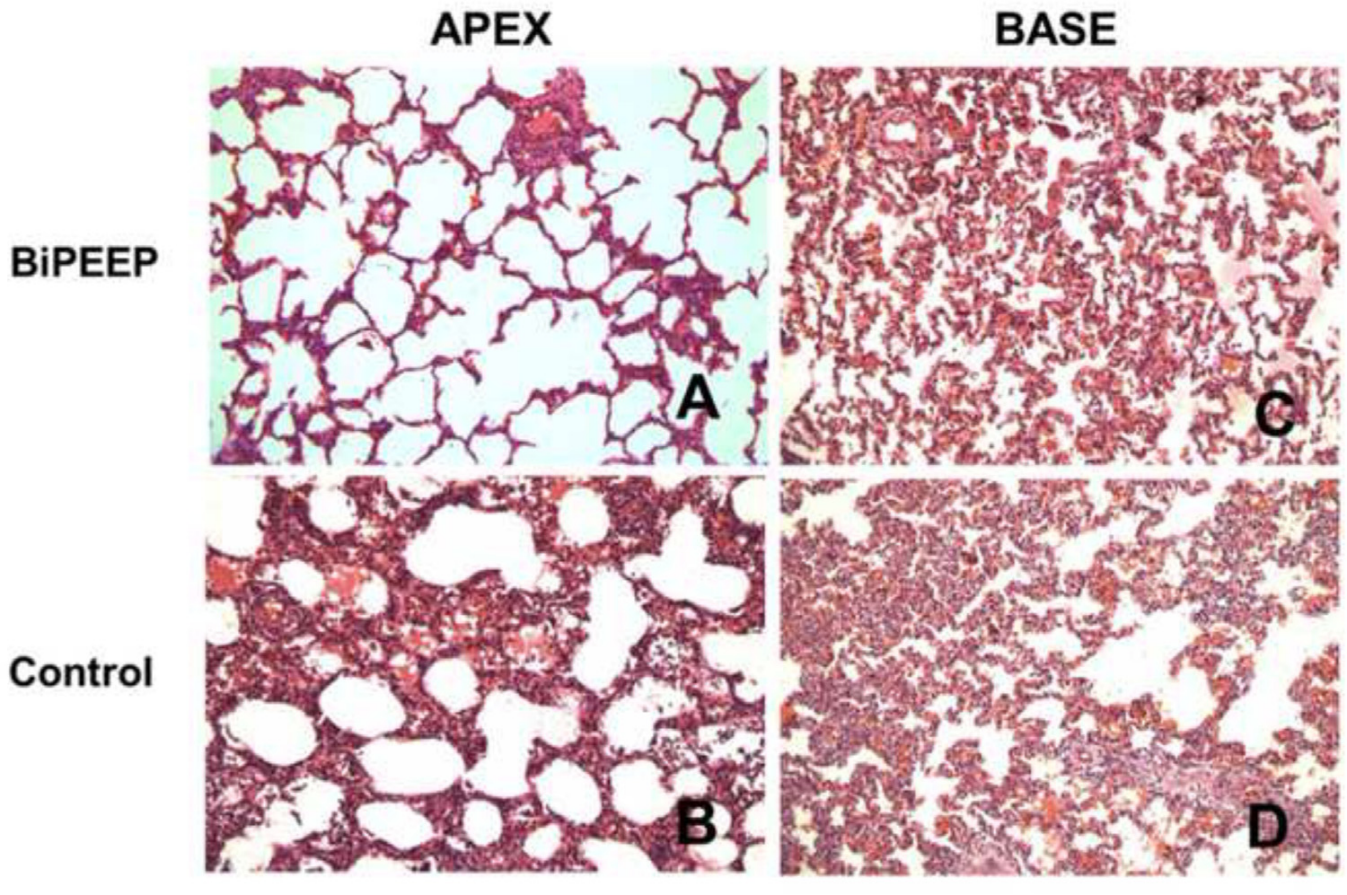
Histopathological analysis (magnification 200 ×) showed cellular infiltrates, thickening of the alveolar septae and atelectasis in the Control group. Both groups presented greater alteration in relation to the base (p < 0.01). However, the BiPEEP group presented less morphological alteration in relation to the control group (p < 0.05). (A) Apex BiPEEP group; (B) Apex Control group; (C) Base BiPEEP group; (D) Base Control group.

The histopathological analysis (Fig. 4) of the control group showed changes in lung morphology characterized by cellular infiltrates, thickening of the alveolar septae and atelectasis, which were attenuated in the BiPEEP group (p < 0.05), whereas both groups presented greater histological alterations in the lung bases (p < 0.01). However, the BiPEEP group presented less morphological alterations then the control group (p < 0.05).

## Discussion

When comparing monotonous ventilation with BiPEEP, the main findings of the study were: 1) A ventilatory strategy with two levels of PEEP resulted in improved gas exchange; 2) The oscillating PEEP showed reduced inflammatory response assessed by IL8; 3) Less structural histological abnormalities when using two levels of PEEP. This study, comparing the two levels of PEEP with conventional ventilation, is the first report in the literature using this ventilatory strategy in an experimental model of ARDS induced by oleic acid.

Variable mechanical Ventilation (VV) attempts to incorporate the physiological basis of spontaneous ventilation during MV and is defined as a ventilatory mode characterized by the oscillation of one or more respiratory parameters. It aims to mimic the variability observed in physiological ventilation and the natural breathing pattern, which changes from cycle to cycle. Several experimental studies reported the beneficial effects of distinct variable ventilation strategies on lung function using different models of lung injury and healthy lungs. Variable ventilation seems to be a viable strategy for improving gas exchange and respiratory mechanics and preventing lung injury associated with mechanical ventilation. However, further clinical studies are necessary to assess the potential of variable ventilation strategies for the clinical improvement of patients undergoing mechanical ventilation.^17^

In this study, the ARDS induced by oleic acid resulted in the reduction of the PaO_2_/FiO_2_ ratio in both groups, as reported by Schuster.^18^ Variable mechanical Ventilation (VV) has been evaluated in experimental studies showing beneficial effects in respiratory mechanics, gas exchange and pulmonary function in animal models with or without lung injury.^17^ However, there are only three clinical studies that have evaluated this approach, and the objectives as well as the outcomes are different in each of these studies.^19^, ^20^, ^21^

When analyzing the respiratory mechanic parameters, we observed a significant increase in airway resistance at 30 minutes after induction in the control group, and no change in the BiPEEP group. Several studies have demonstrated improvement in respiratory mechanics in experimental models of ARDS using VV. A possible explanation may be the use of different changes in PEEP levels during ventilation.^17^ Boker et al.^22^ compared conventional ventilation to VV during 5 hours in a porcine model of ARDS, with VT and RR as non-fixed variables, and demonstrated improvement in respiratory mechanics, gas exchange and reduction of the shunt fraction. This finding may be associated with the VT variability used by the authors, which was generated randomly, thus reaching the critical pressure of alveolar opening, culminating in the opening of non-ventilated lung regions. We demonstrated the presence of hemodynamic changes (HR, PAP and ETCO_2_) only after induction with AO, and this finding was evident in a similar way described by Boker et al.,^22^ who demonstrated a significant increase in hemodynamic variables immediately after induction of lung injury, remaining at high levels during the experiment.

We demonstrated that ventilation using two levels of PEEP improves oxygenation as well as reduces PaCO_2_. These findings corroborate with several experimental studies in ARDS that demonstrated improvement in gas exchange with VV.^11^^,^^22^, ^23^, ^24^, ^25^, ^26^, ^27^, ^28^ The beneficial effects of variable mechanical ventilation reported in this study are similar to those previously described by Lefevre et al. and Mutch et al. However, in these studies, the respiratory rate was randomly varied at a given value and VT was adjusted to keep minute ventilation constant during oleic acid-induced lung injury or unilateral lung collapse model. In both cases, a single level of variability in mechanical ventilation was applied and resulted in a significant increase in lung compliance and PaO_2_ in comparison with conventional ventilation. Nam et al. demonstrated that variable ventilation had no beneficial effects over conventional ventilation. This observation suggests that factors other than variability *per se* can be important for conferring beneficial effects on lung physiology in this setting.

A possible factor responsible for the effectiveness of BiPEEP regarding gas exchange is the effect related to alveolar recruitment and derecruitment. This is evident in the study by Ma et al. whose hypothesis was tested in a computational pulmonary model. The model also showed that recruitment/derecruitment dynamics contributed to a relative efficacy of variable ventilation, providing lung units open faster than close, since the critical opening or closing pressure threshold has been crossed. We conclude that the dynamics of recruitment and derecruitment in the lung may be important factors responsible for the benefits of VV compared to CV.

In our study, we observed a significant reduction in IL-8 levels in the BiPEEP group. Unfortunately, the same finding was not observed regarding IL-17a. Aberrant IL-17 signaling can lead to an exacerbate inflammation, which can result in a harmful cellular damage. Acute Respiratory Distress Syndrome (ARDS) can develop following traumatic injury and/or a major inflammatory episode, such as sepsis, and is characterized by severe lung dysfunction, fluid accumulation (edema), hypoxia, and excessive neutrophil infiltration/activation.^22^ Boker et al. observed that the concentration of interleukin-8 (IL-8) in the tracheal aspirate after 5 hours of VV was lower compared to conventional mechanical ventilation, although with similar findings in pulmonary edema.^22^ Corroborating these observations, Arold et al.^11^ demonstrated that, after 3 hours of VV in guinea pigs without lung injury, there was a reduction in the concentrations of IL-6 and Tumor Necrosis Factor alpha (TNF-α) in bronchoalveolar lavage.

Thammanomai et al.^4^ investigated the physiological consequences of variable mechanical ventilation in a mouse model of ALI and showed to be superior to conventional ventilation in f lung mechanics and injury biomarkers. The possible explanation for the beneficial effects of VV lies in the use of a non-linear system, which is similar to the biological variability of the respiratory system. These models may increase VT based on the nonlinear characteristics of collapsed^8^ and normal^9^ alveoli. Thus, there are two main reasons for improvement of pulmonary function during VV, recruitment and stabilization of lung regions, enhancing gas exchange, and improvement in ventilation-perfusion ratio.

This study is the first in the use of PEEP as a variable to be modified during VV. However, it presents limitations such as the fact that it is an experimental study which cannot be directly transposed to clinical practice, where the use of variable PEEP could not titrated and this tool is not implemented in all commercially available ventilators. We decided to use PEEP 5 cm H_2_O based on clinical observation, where in many centers this is the initial setting PEEP during ventilation, and we aimed to double this value in order to maintain aeration and avoid hyperdistension.

Our study showed that mechanical ventilation with bi-oscillatory levels (BiPEEP) improved ventilatory mechanics and gas exchange, in addition to reducing inflammatory markers and protecting the lungs, resulting in less histological damage.

## Conflicts of interest

The remaining authors have disclosed that they do not have any conflicts of interest.
